# Telling the story of the opioid crisis: A narrative analysis of the TV series *Dopesick*

**DOI:** 10.1371/journal.pone.0301681

**Published:** 2024-04-04

**Authors:** Joel Piqué-Buisan, Josep-E Baños, Irene Cambra-Badii

**Affiliations:** 1 Faculty of Medicine, Universitat de Vic–Universitat Central de Catalunya, Vic, Spain; 2 Observatory of Humanities in Medicine, Hospital d’Olot i Comarcal de la Garrotxa Foundation, Olot, Spain; 3 Research group on Methodology, Methods, Models and Outcomes of Health and Social Sciences (M3O), Faculty of Health Sciences and Welfare, Center for Health and Social Care Research (CESS), Universitat de Vic–Universitat Central de Catalunya, Vic, Spain; 4 Institute for Research and Innovation in Life Sciences and Health in Central Catalonia (IRIS-CC), Vic, Spain; Purdue University, UNITED STATES

## Abstract

*Dopesick* (2021) is the first TV series whose plot deals exclusively with the opioid crisis in the United States. The current study uses narrative analysis and framing theory to explore this series, discussing its portrayal of the people and themes involved in the opioid crisis. Our analysis found that although *Dopesick* attempts to portray multiple dimensions of the opioid crisis, its narrative oversimplifies the story in attributing the cause of the problem almost exclusively to Purdue Pharma and its director Richard Sackler, while downplaying other factors that contributed to the opioid crisis. Thus, the narrative in this TV series tends to offer simple explanations to a complex problem for which simple solutions are likely to be inadequate.

## Introduction

The opioid crisis is one of the most severe public health crises in recent U.S. history [1, 2]; it was declared a public health emergency in 2017 [3]. The latest World Drug Report [4] highlights the importance of opioids in illegal drug use. In 2020 alone, overdoses of opioids resulted in 68,630 deaths in the U.S., accounting for 74.8% of all drug overdose deaths [5].

Aiming to improve pain management and alleviate pain-associated suffering, physicians started to prescribe opioids more often in the 1990s [6–8]. However, strong opioid analgesics were sometimes used to treat patients who did not actually need them [6]. Overprescribing is but one factor among many that contributed to the opioid crisis, which developed through a complex network of agents, including patients, doctors, drug companies, and even the healthcare system and government drug regulatory offices [1, 2, 9–11].

The complexity of the opioid crisis and the multiple interests and viewpoints involved result in “opioid storytelling” from a wide variety of different perspectives [12]. Journalistic approaches portrayed the opioid crisis in books such as *Pain killer*: *an empire of deceit and the origin of America’s opioid epidemic* [13], *Dopesick*: *dealers*, *doctors and the drug company that addicted America* [14], and *Empire of pain*: *the secret history of the Sackler dynasty* [15].

Likewise, in recent years several audiovisual documentaries have also focused on the opioid crisis, and fiction series, especially medical dramas, have also included opioid addiction in some episodes [16–23]. However, portrayals of the opioid crisis in fiction series have been limited to a single dimension such opioid use disorder and the difficulties of recovery, pharmaceutical companies’ responsibilities, or increased criminality associated with drug addiction.

*Dopesick* [24] is the first drama miniseries whose plot deals exclusively with the opioid crisis and includes multiple dimensions in its portrayal. Based on the non-fiction book *Dopesick*: *dealers*, *doctors*, *and the drug company that addicted America* [14], the TV series portrays the opioid crisis in the U.S., focusing on the production, marketing, sales, and consumption of OxyContin^®^.

Previous studies have looked at how the media and social media depict the opioid crisis [25–29]; a consistent conclusion is that there is an emphasis on the use of personal, emotionally touching, and often stereotyped stories at the expense of in-depth thematic coverage. However, only a few studies with a narrow scope published in academic and opinion journals have explored its depiction in recent TV shows [30, 31].

The current study aimed to use a qualitative approach to explore and analyze the narrative in the TV series *Dopesick*, focusing on how the opioid crisis is portrayed. Rather than carry out a classical content analysis, we decided to perform a theme-based inquiry following the model proposed by Riessman [32]. We focused our analysis on the contents of the story, in other words, on the elements selected for portrayal and their salience. We sought to determine which elements, characters, and narrative arcs are predominant in the plot.

Therefore, our first two research questions are descriptive and investigate the way *Dopesick* tells the opioid crisis on its narrative:

RQ1: Who is involved in the opioid crisis as it is portrayed in *Dopesick*?
RQ2: Which narrative themes are prominent in *Dopesick*, and how are they presented?

Furthermore, we sought to analyze the fidelity of the narrative according to Fisher’s narrative paradigm [33]. Fisher argued that narrative coherence and fidelity provide insights into why some stories can be accepted and others cannot [34]. Whereas coherence refers to how well the story fits together in terms of details, characters, and events, fidelity refers to the “truth qualities” of the story [33]. Considering that the opioid crisis is a real public health crisis, we chose to evaluate the fidelity of *Dopesick*’s narrative by comparing the information presented in the series with reports on the opioid crisis in the scientific literature through a third research question:

RQ 3: To what extent does *Dopesick’s* narrative agree with the information in the scientific literature?

### Theoretical framework and methodological approach

All approaches to narrative analysis explore stories through their narrative arcs, plots, and motives [32, 35]. Especially in TV series, characters, institutions, and storylines are important and can be used specifically to persuade or entertain an audience [36]. The way TV series portray complex, real problems in their fictional narratives is of particular interest. Media can tell audiences not only which issues to consider, but also how to think about them [37].

Framing theory offers a comprehensive framework for organizing and managing information in everyday life [38]. Basically, frames are persistent, shared organizing principles that work symbolically to structure the social world [39]. Gitlin [40] characterized framing as the principles involved in the selection, emphasis, and presentation of narrative elements that reflect implicit theories regarding the existence and significance of events. Framing selects aspects of perceived reality that serve to “*define problems*—determine what a causal agent is doing with what costs and benefits, usually measured in terms of common cultural values; *diagnose causes*—identify the forces creating the problem; *make moral judgments*—evaluate causal agents and their effects; and *suggest remedies*—offer and justify treatments for the problems and predict their likely effects” [41].

Framing theory underscores the importance of two key elements: selection and salience [41]. This theory posits that any given issue can be examined from multiple perspectives, each emphasizing distinct implications for various values or factors; and this process involves the deliberate selection of specific facets of reality and elevating their prominence within a communication text. Gamson and Modigliani [42] formulated a notion of frames as “a central organizing idea or story line that provides meaning to an unfolding strip of events. The frame suggests what the controversy is about, the essence of the issue”.

Although framing theory originated in attempts to explain how individuals interpret and understand reality in the fields of communication and sociology [38, 41–44], this approach has also been employed in health communication research. Framing has been useful in analyzing how health information is presented in the media and how different ways of presenting health information can affect audiences [45–47]. Studies focusing on “media frames” are those that analyze how frames are presented, while studies focusing on “audience frames” analyze the impact the frames have on the audience, typically in the short term [37].

The methodology within this framework has yet to be fully standardized [47]. Health communication studies have applied media framing through diverse methodologies: for example, Wang and Parris [48] combined framing theory with narrative analysis to examine the risks associated with the depiction of teenage suicide in the TV series *13 reasons why*. Framing theory was combined with quantitative content analysis by Kim and Willis to analyze the American news media’s portrayal of individual and societal responsibility in obesity [49] and by Van den Bulck et al. [50] to analyze the use of alcohol in the prime-time American youth TV series *The OC*. However, these analyses lack a unified methodology, and the bridge between framing theory and content analysis, especially qualitative approaches, is not always clear.

Since the presentation of a narrative invariably involves adopting a perspective or frame, we aimed to align the three research questions presented above with framing theory by considering a fourth research question:

RQ 4: How is the opioid crisis framed in *Dopesick*?

## Materials and methods

### Sample

The present study analyzes the narrative told through the eight episodes of *Dopesick*. Each episode is about one-hour long and contains an average of 57 scenes (Table 1). Each scene is framed in a certain time and location; changes in either of these coordinates signal a change to a different scene. According to this definition, we analyzed 458 scenes.

**Table 1:** *Dopesick* series summary by episodes.

Episode #	Title	Duration (hours and minutes)	Number of scenes
1	First Bottle	1:02	57
2	Breakthrough Pain	1:02	65
3	The 5th Vital Sign	0:57	39
4	Pseudo-Addiction	1:01	41
5	The Whistleblower	1:03	61
6	Hammer the Abusers	1:00	64
7	Black Box Warning	1:02	51
8	The People vs. Purdue Pharma	1:05	80
Total		8:12	458

### Coding procedure and analysis

Following the approaches used in recent narrative analyses of TV series [48, 51], we sought to “take apart the logic of the stories to determine their meanings” [33]. Using scenes as the unit of analysis, two researchers analyzed the content independently, while a third one supervised the whole process. The two independent analyses were compared, and discrepancies or disagreements were settled through discussion with the third researcher. The results were merged into a single dataset.

To address the question of who was involved in the opioid crisis according to *Dopesick* (RQ 1), we followed these steps. Each of the two researchers independently watched the entire series to identify characters, record actions, delineate plots, reconstruct timelines and identify salient elements, noting the year of the event depicted and the characters involved in each scene. To determine the year of the event depicted in the scene, we used extradiegetic notes in the episodes (i.e., the display of the year as a number in the transition to the scene). In the few cases where no extradiegetic signal was present, we relied on cues of scene continuity provided by characters’ clothing and the settings where the scene took place to determine the year in which the event occurred. Then, we grouped the characters by their affiliations (e.g., with Purdue Pharma or U.S. Attorney’s office) or main role in the narrative (e.g., physician or addicted patient). It was especially challenging to categorize physicians for analysis because of their pervasive presence across all categories; in addition to acting as clinicians prescribing pain medication to patients, some medical professionals were associated with Purdue Pharma and its sales, were witnesses in the trials, or became addicted themselves. Thus, although David Haddox and Russel Portenoy were both medical physicians, they were grouped according to their affiliation with the pharmaceutical company, rather than as clinical physicians, because they both promote OxyContin^®^ at scientific meetings and in the media, and Haddox was hired by the Purdue Pharma. In analyzing the character Samuel Finnix, we considered the scenes related to his role as a physician separately from those related to his addiction and recovery.

To determine the prominent themes in *Dopesick* and explore how they are developed (RQ 2), we took detailed notes about the key narrative elements such as character development, character interactions, and recurring themes. We developed a system to categorize and code the narrative elements as themes. Themes were not predefined; rather, they emerged from the analysis as the data accumulated. Again, two researchers reviewed each episode independently and abstracted data. To keep subjectivity in this classification to a minimum, two coding rounds were needed. To classify themes, we considered the characters present in the scene, taking the overarching theme into account.

We decided on three main narrative themes: 1) the government’s investigation, mainly by the U.S. Attorneys’ office and to a lesser extent by the DEA, including the grand jury proceedings; 2) unveiling OxyContin’s^®^ accountability network: the responsibilities of different agents, from the Sackler family to healthcare providers, including Purdue Pharma’s sales agents and the U.S. Food and Drug Administration (FDA) through its approval and labeling; and 3) the impact of the opioid crisis on society explained through the lives of individuals who become substance-dependent after being prescribed OxyContin^®^, including their background stories (e.g., everyday life and work before) and the narratives of their addictions. Then, we counted the number of scenes involving each of these themes to determine their salience.

For each of these narrative themes, we have included a subcategory called “background stories” for scenes that include one or more of the main characters of the storyline articulating the theme without dealing directly with the thematic concept. For example, in Episode 1, scenes showing Betsy Mallum’s life at work are not directly related to her addiction, but they form part of her storyline because they enable viewers to track the development of her character and story.

To analyze the extent to which *Dopesick*’s narrative aligns with reports on the opioid crisis in the scientific literature (RQ 3), we compared information about the opioid crisis in the U.S. presented in the series with information from other sources, especially articles in scientific journals obtained from Pubmed scientific database. As in the content analysis, each of the two researchers independently assessed narrative fidelity in *Dopesick* and then compared results to mitigate subjective discrepancies.

To our knowledge, no published studies have examined the opioid epidemic through the perspective of framing. Thus, to analyze how the opioid crisis is framed in *Dopesick* (RQ 4), we decided to apply an inductive method to analyze the narrative themes identified in RQ 2 to determine which frame(s) underlie(s) the narrative.

## Results

### Exploring the roles of the characters in *Dopesick*

Table 2 reports the frequency of appearance *Dopesick’s* main characters, some of whom are based on real people like the Sackler family, owners of Purdue Pharma. The groups of characters appearing in the most scenes are members of the U.S. attorneys’ offices (n = 198; 43.2%) scenes, followed by employees of Purdue Pharma (n = 164; 35.8%), patients who use OxyContin^®^ and end up becoming addicts (n = 136; 29.7%), the Sackler family (n = 119; 25.9%), health professionals (n = 63; 13.7%), DEA agents (n = 57; 12.5%), and FDA officials (n = 87; 1.9%).

**Table 2:** Frequency of appearance of characters, grouped according to their affiliation, in scenes in the series *Dopesick*.

U.S. attorneys		Purdue Pharma Company		OxyContin^®^ substance-dependent individuals, and their families		Sackler family		Health professionals		DEA		FDA	
Rick Mountcastle *	85	Michael Friedman *	27	Samuel Finnix	55	Richard Sackler *	52	Samuel Finnix	35	Bridget Meyer	47	Karen Moles	5
Randy Ramseyer *	85	Howard Udell *	21	Betsy Mallum	55	Jonathan Sackler *	19	Sister Beth Davies *	10	Jermaine Spellman	5	Cynthia McCormick *	4
John Brownlee *	23	Paul Goldenheim *	13	Elizabeth Ann McClung	14	Raymond Sackler *	14	Art Van Zee *	8	Grant Simmons	5		
Jay McCloskey *	5	Martin Willis	17	Logan Parker	7	Kathe Sackler *	12	Leah Turner	9	Director Melton	3		
		Billy Cutler	41	Marianne Skolek *	5	Mortimer Sackler *	10	Alan Spanos *	1				
		Amber Collins	21			Arthur Sackler *	4						
		Russell Portenoy *	3			Theresa Sackler *	3						
		David Haddox *	8			Beth Sackler *	3						
		Maureen Sara	8			Mortimer Sackler Jr. *	2						
		Drea Price	5										
198		164		136		119		63		57		9	

* Character based on a real person

*Note*: more than one character may appear in each scene.

### Narrative themes in *Dopesick*

Nearly all the events presented in *Dopesick’s* narrative take place between 1996 and 2007. Rather than presenting events in a strictly chronological order, the narrative of the series jumps around in time to focus on different aspects of the story. Thus, each episode includes scenes from different years, and to help viewers place the scene in a particular year, the series normally uses extradiegetic announcements. S1 Annex relates the events in each episode in chronological order.

Of the 458 scenes, 94 (20.5%) are set in 1996, the year in which OxyContin^®^ was launched in the United States, 87 (19.0%) in 2002, the year in which the assistant U.S. attorneys for Virginia (19.0%) opened their investigation, and 54 (11.8%) in 1999, the year in which the DEA opened their investigation.

Of the three main narrative themes, the one that is developed in the greatest number of episodes is the stories of individuals who become substance-dependent after being prescribed OxyContin^®^ (n = 190; 41.5%), followed by the government’s investigation (n = 154; 33.6%) and Oxycontin’s^®^ accountability network (n = 114; 24.9%). Table 3 details the number of scenes that deal with different aspects of each of the three main narrative themes in each episode.

**Table 3:** Number of scenes dealing with each of three main narrative themes in *Dopesick*.

TV series episode	OxyContin^®^ substance-dependent individuals		Government’s investigations				OxyContin’s^®^ accountability network				Total
	*Addictions*	*Background stories*	*DEA*	*U*.*S*. *Attorneys*	*Grand Jury proceedings*	*Background stories*	*Sackler family and Purdue Pharma executives*	*Purdue’ Sales representatives*	*FDA*	*Background stories*	
**1**	0	23	5	5	5	4	4	7	1	3	57
**2**	1	9	7	14	7	4	5	16	0	2	65
**3**	7	6	5	4	2	2	6	6	0	1	39
**4**	15	3	3	9	3	0	4	4	0	0	41
**5**	17	10	6	12	0	3	11	2	0	0	61
**6**	17	20	9	6	4	1	4	1	0	1	63
**7**	20	2	3	6	3	1	8	5	1	2	51
**8**	11	29	2	18	1	0	13	5	0	2	81
**Total**	88	102	40	74	25	15	55	46	2	11	458
**Total for Main narrative themes**	190		154				114				458

#### OxyContin^®^ and the impact on society and substance-dependent individuals’ lives

The most frequently recurring theme in *Dopesick* is the impact of OxyContin^®^ on the lives of individuals who became substance-dependent and on their families. Although these stories are heterogeneous, they share some elements. Samuel Finnix, Betsy Mallum, and Logan Parker all started using OxyContin^®^ in 1996 when they were prescribed the drug. Only young Elizabeth Ann McClung first uses OxyContin^®^ recreationally, snorting a pill at a party in 1997. Samuel Finnix is the only one who starts treatment with a dose of 20 mg rather than 10 mg, as a consequence of Purdue Pharma’s “individualize the dose” marketing campaign. Betsy Mallum’s dose is increased as a result of Purdue Pharma’s “breakthrough pain” marketing campaign (5 scenes). Both Finnix’s and Mallum’s addictions spiral after self-medication. The series shows how patients became dependent on OxyContin^®^ and suffer withdrawal syndrome (13 scenes), which consists of the same symptoms and signs in all cases: drowsiness, hallucinations, sweating, itching, and tics.

Dependent individuals will go to great lengths to obtain the drug. The series shows how patients obtain OxyContin^®^ legally in hospitals and private medical clinics (11 scenes) as well as in pain clinics of questionable medical ethics (4 scenes). It also shows how substance abusers obtain the drug illegally. Walt, a local drug dealer, sells pills to both Betsy Mallum and Samuel Finnix (5 scenes), and one of the attendees at a community meeting to help addicts recover sells OxyContin^®^ in the restroom (1 scene). Samuel Finnix looks for medication in different states, steals medication belonging to his patients, asks a Purdue sales representative for samples, and hides medication in his house and in his office (6 scenes).

Desperate actions to obtain drugs include prostitution, which is depicted in specific scenes from the first episode, where Elizabeth Ann McClung offers to have sex in exchange for money in a parking lot (1 scene). A doctor in a pain clinic suggests that Betsy Mallum can pay for her consultation with sex (1 scene); although she refuses his advances, she does agree to have sex with a pawnshop employee in exchange for more money when she sells her mother’s family jewels (1 scene).

The series shows how many individuals who were prescribed OxyContin^®^ eventually switched to cheaper, more easily obtainable opioids when their access to OxyContin^®^ was restricted. Betsy Mallum’s first tries heroin in 2000 and goes on to die of an overdose in 2002 (2 scenes). Betsy uses heroin in derelict areas with high concentrations of drug users.

*Dopesick* also shows various approaches to overcoming OxyContin^®^ addiction. Betsy Mallum undergoes three different types of treatments: she attends a church-based support group for addicts (2 scenes), is admitted to a rehabilitation clinic where she is tied to a bed (1 scene), and she is subjected to exorcism-like ceremonies at the community church (2 scenes). After Samuel Finnix’s addiction leads to malpractice and eventually to his medical license being revoked after he botches a routine minor surgery (4 scenes), he is forced to enter a rehabilitation clinic for 90 days, where he undergoes group therapy (9 scenes). After he is discharged, Finnix undergoes treatment with methadone at Dr. Art Van Zee’s clinic (8 scenes) and psychotherapy with Sister Beth Davies (2 scenes). Later, the methadone treatment is replaced with Suboxone^®^ (3 scenes). Finnix also arranges for Logan Park and Elizabeth Ann McClung to undergo methadone treatment with Dr. Art Van Zee and personally transports them to the clinic (9 scenes).

Families are unevenly represented in the narratives of addictions. Only Betsy Mallum’s family is portrayed in any depth (34 scenes). Betsy’s family is representative of the town’s inhabitants: they are poor, hard-working, religious, and have difficulties accepting their daughter’s homosexuality. When Betsy becomes addicted, she initially refuses their help. She argues regularly with her parents and steals all the family jewelry to pawn for drug money, bringing additional hardship on the family. Episodes 7 and 8 show Betsy’s mother participating in demonstrations at a museum to denounce the Sacklers, meeting with Dr. Art Van Zee and Sister Beth Davies, and attending the Abingdon Court trial. Another character, Maryanne Skolnick, based on a real person, also represents family involvement, but her role is limited to the process of seeking justice after her daughter’s death (6 scenes).

#### The government’s investigation

This theme comprises the stories of the characters working at the U.S. Attorney’s office and at the DEA. The U.S. Attorney’s office’s investigation shows the manipulation of the promotional video "I got my life back" (13 scenes); Purdue Pharma’s subsidization of networks purporting to be independent that recommended the use of OxyContin^®^, such as the American Society of Pain, the Academy of Pain Medicine, and patients’ associations such as the National Foundation for the Treatment of Pain and the American Chronic Pain Association (8 scenes); and suspected corruption in Purdue Pharma’s hiring of former government officials (FDA’s Curtis Wright, 6 scenes; and the U.S. Attorney for Maine, Jay McCloskey, 6 scenes).

Regarding grand jury proceedings, two preliminary court hearings that take place in 2003 are represented in Episode 3. In the first, U.S. Attorneys Rick Mountcastle and Randy Ramseyer introduce the promotional video “I got my life back” as evidence that Purdue Pharma “manipulated basic facts about the drug” by claiming that it was not addictive, alleging that the participants were deceived when they were recorded for the video. At the second hearing, the U.S. Attorneys provide evidence that the American Pain Society, which claimed to be an independent medical group, received significant funding from Purdue Pharma.

In Episode 8, court proceedings have a more prominent place in the narrative, where a trial set in 2004 and 2005 accounts for 15 scenes. These scenes include witnesses’ testimony about how Purdue Pharma promoted OxyContin^®^, the effects of its use and withdrawal from the drug, and the ease of access to the drug. One trial takes place in 2007 in Abingdon, Virginia federal courthouse (7 scenes). The U.S. attorneys bring charges against Purdue Pharma executives, and viewers see testimony from people whose relatives died from OxyContin^®^, often addressing the executives directly. The judge accepts the $600 million settlement, but upholds the plea bargain by which the executives would only be convicted of misdemeanors and sentenced to three years’ probation.

The DEA investigation, in the TV series led by the fictional character Bridget Meyer, began in 1999. In 20 scenes, she alerts her superiors about the increases in overdoses and crime associated with the consumption and diversion of OxyContin^®^ and confronts Purdue Pharma and the FDA through press conferences to inform the general public about these problems. The tension between the DEA and the FDA over the OxyContin^®^ label occupies a large part of this narrative axis (12 scenes). When promoted to become the Deputy Director of the DEA Diversion Area, Meyer goes to the FDA to request restrictions on the distribution of OxyContin^®^ (5 scenes); she also meets with Purdue Pharma executives in the DEA’s offices in 1999 and 2001 (2 scenes). Faced with the FDA’s and Purdue Pharma’s refusals to change the labeling of the drug, Meyer’s DEA team undertakes a forensic investigation to provide evidence that many overdoses occurred among people who were prescribed OxyContin^®^ and used it exactly as indicated on the label (6 scenes). However, neither Purdue nor the FDA accept the evidence.

#### OxyContin’s^®^ accountability network

The third major narrative theme of *Dopesick* traces the responsibility of different actors in the opioid crisis. From the very first episode, the FDA’s original labeling of OxyContin^®^ is presented as a major problem, epitomized in Bridget Meyer’s statement *“That damn label caused it all”*. Curtis Wright, a former U.S. government official who played an important role in approving OxyContin^®^ during his tenure at the FDA, is singled out as a major culprit (10 scenes). Just two years after the drug’s market release, Wright was recruited to serve as Purdue Pharma’s Executive Director of Medical Affairs. In Episode 2, when the U.S. prosecutors question Wright’s role in the FDA’s accepting that OxyContin^®^’s addiction rate was < 1% in approving the drug’s label in the light of his career move to Purdue Pharma, FDA officials respond that many people leave government employment for more lucrative jobs in private industry, and that this is not illegal and cannot be considered corruption because it is just how the system works. In Episode 7, entitled “Black Box Warning”, viewers see how the FDA finally agrees to change the OxyContin^®^ label by adding a black box warning. However, the wording of the warning is crucial. Because it merely states that “addiction is reported to be rare”, the new label continues to allow OxyContin^®^ to be prescribed for moderate pain; moreover, it also states that the drug can be used even "for an extended period of time". Thus, Richard Sackler and Purdue Pharma executives actually consider the change in the label to be a boon rather than the detriment they had feared.

Purdue Pharma’s organizational structure is portrayed as strongly hierarchical. The focus remains on Richard Sackler, who meets with his family to discuss financial issues at his house or in the Sackler Wing of the Metropolitan Museum of Art in New York City (11 scenes). Meetings with top executives (Michael Friedman, Howard Udell, and Paul Goldenheim) are held in Purdue Pharma’s offices (24 scenes). Top management’s decisions are relayed directly to sales executives and implemented in training sessions for sales representatives (18 scenes). Viewers see how the sales representatives promote OxyContin^®^ to doctors and how they are motivated to sell more through a system of awards and financial compensation (20 scenes). Although the series lays responsibility on Richard Sackler by emphasizing his leadership and his speeches (e.g., through the narrative of pushing for OxyContin’s^®^ introduction in Germany, 5 scenes), it is Purdue Pharma’s executives rather than the Sackler family who take the blame, as is shown in the Episode 8 through both fictitious (8 scenes) and documentary images (19 scenes).

Physicians play an important role in the company’s strategy through their prescribing and promoting the drug. The series portrays the intricate web of connections between sales representatives and healthcare professionals, involving gifts and bonuses associated with prescribing OxyContin^®^. *Dopesick* assigns a minor role to one physician based on a real person, Dr. Alan Spanos (3 scenes), who unscrupulously promotes OxyContin® for financial gain. By contrast, one of the major roles in the series, the fictional character Samuel Finnix, is a dedicated physician in a small town in Appalachia who is duped by a Purdue Pharma sales representative into prescribing OxyContin^®^ to treat his patients. When Finnix himself is prescribed OxyContin^®^ after an automobile accident, he develops a substance-abuse disorder; after a tumultuous journey to recovery, Finnix is redeemed by dedicating his life to helping others overcome their addictions.

Another physician based on a real person is Art Van Zee, an activist fighting against the opioid epidemic in Appalachia. In *Dopesick*, Van Zee is portrayed as a physician who cares for his community, addressing the issue of addiction not only within Purdue Pharma but also by treating individuals with substance use disorders (8 scenes). In this capacity, he helps Samuel Finnix to recover from his addiction to OxyContin^®^ through treatment with methadone and Suboxone^®^, a fixed dose combination of buprenorphine and naloxone.

### Analysis of narrative fidelity in *Dopesick*

Regarding narrative fidelity, the temporal and spatial setting appears to be accurate and faithful. According to the scientific literature, the number of prescriptions for OxyContin^®^ increased from 670,000 in 1997 to 6.2 million in 2002 [52], thus attesting to the explosive growth depicted in this time period in the *Dopesick* narrative. Moreover, Appalachia, where the impact of the crisis is shown in the series, was indeed the geographical region with the highest concentration of OxyContin^®^ abuse in that period [53], and central Appalachia was an early focal point in the opioid epidemic [54]. In this largely rural area, many people working in physically demanding industries such as coal mining, agriculture, and logging were vulnerable to prescription opioids’ promise of pain relief [55, 56].

The composite portrait of substance abusers in the series is in line with the scientific literature, which indicates that people of all ages, sexes, and socioeconomic backgrounds abuse opioids, especially in rural settings [9]. The characters mostly adhere to an initial stereotype of people from Appalachia, coming from rural areas, with a low level of education, great gender inequalities, and a high level of crime. Some scholars have speculated that these social stereotypes have been created by economic and political forces to justify the exploitation of Appalachian peoples through industrialization and natural resource extraction [57–59]. The fact that all the substance abusers in the series are Caucasians could be thought to reinforce the interpretation of the opioid crisis as a “white disability” [12], but this portrayal truthfully reflects the demographic composition of the Appalachian population. Likewise, the path to addiction depicted in the series, where three of the four cases begin with doctor-prescribed opioid use, is in line with figures from the scientific literature, which indicates that 80% of opioid abusers were prescribed opioids before becoming addicted [60]. *Dopesick’s* narrative does not place much importance on the social determinants and contexts of opioid use; this portrayal is not totally discordant with the scientific evidence on the role of economic conditions in driving drug misuse and overdoses, as different studies have reported discrepant results [61–63]. However, the inadequate access to detox treatments and interdisciplinary approaches shown in Betsy Mallum’s story are in line with reports in the scientific literature [64–66]. Moreover, Betsy’s dependence on Oxycontin^®^ leading to heroin addiction reflects a widely reported occurrence [6, 67], and although Betsy eventually dies of an overdose, the incidence in of deaths from heroin overdoses in the series is much lower than in reality [68].

By contrast, *Dopesick*’s opioid storytelling repeatedly emphasizes the inappropriate marketing of OxyContin^®^. By focusing on this aspect, the narrative blames the opioid crisis on Purdue Pharma and its executives, singling out Richard Sackler. The series shows how their responsibility extends far beyond Sackler’s role as the originator and driving force behind the OxyContin^®^ sales system, underlining the financial links between Purdue Pharma and scientific and patients’ societies and highlighting the role of some pain experts and scientific societies as influencers in a clear demonstration of conflict of interest. This portrayal is in line with reality. Prominent pain societies in North America did indeed support the expanded utilization of opioids for treating chronic pain, and they published consensus statements advocating for the supervised and careful use of opioid therapy in patients with chronic pain [69]. This model underlies the government and DEA’s focus on criminalizing drug use [70] which is addressed only limitedly in the series.

The series also shows the roles of medical doctors and scientists (real characters Russell Portenoy and David Haddox) in defending the use of opioids as well as their major direct or indirect conflicts of interest through pain societies financed by Purdue Pharma; this portrayal is grounded in different court records [53, 71–75].

Importantly, however, Purdue Pharma was not an isolated case or solely responsible for the opioid crisis as might be surmised from the information presented in the series. First, it is not the only company that has been found legally responsible in the opioid crisis. In 2022, Johnson and Johnson and the three biggest U.S. drug distributors—Cardinal Health, McKesson, and AmerisourceBergen—ended America’s biggest multi-state legal settlement with a $26bn payout [76].

Moreover, the complex network of responsibilities also includes the FDA and the government. The role of the FDA can be considered one of the most problematic issues portrayed in the series. This portrayal is in line with Makhinson et al.’s [72] conclusion that the influence of experts and scientific societies was second only to that of the pharmaceutical companies and government regulators. Manchikanti et al. [73] consider that the FDA’s uncritical approval of OxyContin’s^®^ label made a substantial, albeit unintentional, contribution to the opioid crisis. Nevertheless, *Dopesick* fails to define the FDA’s responsibility.

Finally, the series fails to mention some facts that would likely interfere with the storyline. For instance, the correlation between the number of opioids prescribed and the extent of non-medical use of opioids or opioid addiction is not straightforward: Singer et al. [77] reported that although the number of prescriptions decreased after 2012, overdose deaths increased. Moreover, no mention is made of other products like fentanyl, Percocet^®^, Percodan^®^, or tramadol that are also associated with the opioid crisis [7, 73, 78–80].

### Framing *Dopesick*

From the start, *Dopesick* makes viewers aware that the consequences of the way that OxyContin^®^ was marketed include misuse, prostitution, and death. The narrative makes it clear that substance abusers are not to blame for their woes, despite Richard Sackler’s attempts to shift the blame onto them. The four characters who come to be addicted to OxyContin^®^ are portrayed as good people who are unknowing victims acting in good faith: they suffer through withdrawal syndrome, try to rehabilitate themselves, seek solutions to their problems, and sometimes succeed in redeeming themselves.

*Dopesick’s* narrative is diametrically opposed to the discredited but still prevalent “moral model of addiction” that characterizes addiction as a manifestation of willpower weakness, suggesting that substance abusers experience an uncontrollable urge to use psychoactive substances and eventually lose the ability to manage their usage despite adverse repercussions like loss of employment, disengagement from or conflicts within personal relationships, difficulty maintaining housing, and health problems [69]. The trajectories of the substance abusers and the challenges they face with their families in *Dopesick* are portrayed in a way that elicits empathy. Moreover, the portrayals of programs based on the moral model (e.g., Betsy’s church) also reflect the consensus in the scientific community.

Our theoretical framework about framing shows that some identities and choices are privileged in the narrative, and others are negated or stigmatized [81]. *Dopesick* devotes several scenes to showing patients becoming substance abusers, but none showing patients benefitting from the treatment, except in the “biased” materials Purdue Pharma and the pain societies they control show to push the drug on society. Thus, the question *What do we do with people who are in pain*? is still open [82].

*Dopesick*’s narrative only partially tackles physicians’ responsibility in prescribing OxyContin^®^. Viewers learn different aspects of this responsibility in courtroom scenes and scenes related to the U.S. Attorney’s investigation, but the healthcare professionals portrayed in these scenes are minor characters and their ethical responsibility is largely unexplored. The exception is Samuel Finnix, whose story is central to the plot. Dr. Finnix is depicted as a competent, dedicated professional who cares deeply for his patients. Finnix is misled by the industry, eventually going from prescribing OxyContin^®^ to becoming a substance abuser himself. After hitting bottom, Finnix seeks redemption by concentrating all his efforts on helping the victims of the opioid crisis. This portrayal provokes empathy, and the drug company’s deception exonerates the physician from blame.

In summary, the prominent element of this narrative is its characterization of the processes resulting in the development of dependence on OxyContin® and the individuals who suffer from it. Some characters’ identities and choices are shown in a favorable light while those of others are condemned [81]: there are obvious “good guys” (e.g., the prosecutors Rick Mountcastle and Randy Ramseyer, both based on real people, and Samuel Finnix and Betsy Mallum, who are fictional inventions) and “bad guys” (most notably, Richard Sackler). Returning to Entman’s definition [41], this frame defines a problem (the opioid crisis in the United States) by identifying a primary causal agent (Purdue Pharma, and particularly Richard Sackler), diagnoses a cause (greed), makes moral judgments (blaming Richard Sackler and his accomplices: sales executives, the FDA, and physicians prescribing opioids and exonerating substance-dependent individuals as victims of inadequately informed medical prescription), and suggests remedies (withdrawing OxyContin^®^ from the market). To relate this narrative, *Dopesick* uses the classic protagonist-antagonist format [83]; however, rather than pitting individual protagonists and antagonists against one another, prosecutors and people harmed by the crisis face off against Purdue Pharma, characterized, as in the title of the book series is based on, as *the drug company that addicted America*.

## Discussion

### *Dopesick*’s narrative about the opioid crisis

Based on real events reported in Beth Macy’s bestselling book *Dopesick*, the eponymous TV series uses a complex narrative involving different groups of characters including businessmen, prosecutors, doctors, and patients to portray the opioid crisis in the United States as a multifactorial problem. This complexity is also reflected in the narrative approach. Each episode comprises scenes from different years to construct the narrative, showing that the causes and consequences are not linear and underlining the interconnectivity of characters and actions and the complexity of the problem. Although many viewers know the outcomes of the characters based on real individual’s stories before watching the series, the outcome of the fictional characters remains a mystery and generates suspense. This suspense is reinforced by the predominant role of the prosecutors’ investigation, which allows viewers to know how the real-life story was developing behind the scenes.

Despite the shifting timeframes within episodes, the bulk of the series takes place in Appalachia between 1996 and 2007. This period comprises the time from the FDA’s approval of OxyContin^®^ in 1995 to Purdue Pharma’s pleading guilty to criminally misbranding the drug and misrepresenting its risks of addiction.

It is interesting to reflect on the disparity between the frequency of appearance of characters and of the narrative themes. In the analysis of the groups of characters, the U.S. attorneys appear most frequently, followed by characters associated with Purdue Pharma, and lastly, OxyContin^®^ substance-dependent individuals and their families.

In contrast, in the analysis of *Dopesick*’s narrative themes, the order is reversed. Although the investigations by the U.S. Attorney’s office and the DEA are central to the plot, *Dopesick* is not primarily a legal drama. The theme that appears in the most scenes is the impact of the crisis on substance-dependent individuals and their families, followed by the Government’s investigation, and finally the network of responsibilities related to OxyContin^®^. The preeminence of the impact on substance-dependent individuals, their families, and society as a whole, underlines the suffering caused by the opioid epidemic.

The analysis of *Dopesick’s* narrative reveals that the geographical, temporal, and multidimensional approach taken by the series towards the opioid crisis primarily emphasizes the responsibilities of Richard Sackler and Purdue Pharma. It provides limited consideration of the duties of the FDA, while downplaying the responsibilities of the government and even the DEA. It is striking that the characters working for the FDA and those working for the DEA (except Bridget Meyer) are not developed further in the TV series. The FDA’s position and actions related to the authorization of the OxyContin^®^ label and of the phrases and graphs used by Purdue Pharma’s sales representatives border on corruption. Finally, *Dopesick* fails to deal with other elements and characters involved in the opioid crisis: there is no mention of the government’s failure to act by creating laws to stop the crisis, the collapse of the healthcare system, other pharmaceutical companies or opioid distributors, or the press. In summary, the TV series *Dopesick* frames the opioid crisis in a way that identifies Purdue Pharma (and in particular, Richard Sackler) as the primary causal agent, morally condemning the company and its chief executive and suggesting that withdrawing OxyContin® would be a step toward resolving the crisis.

### Methodological, theoretical, and practical implications

Given the complexity of the series *Dopesick*, we were obliged to employ a complex inductive approach requiring multiple revisions to harmonize criteria and avoid subjectivity. This process elucidated the narrative structure in a manner that we had not initially envisioned. Our approach allowed us to compare and contrast the narrative themes we identified together with the portrayals of the characters in the series to help us understand the themes included in the narrative and their salience.

Applying Fisher’s narrative paradigm to determine *Dopesick’s* fidelity vis-à-vis the scientific literature enabled us to analyze another important dimension of the series and provided useful data leading to new insights. We considered it unnecessary to use this paradigm to analyze narrative coherence because our analysis of scenes and characters in exploring narrative themes yielded ample results. The lack of empirical measures and standardized analytical methods for the theoretical constructs in Fisher’s narrative analysis would have made this approach challenging [33]. Therefore, we looked to framing theory to provide an additional theoretical framework to guide our approach.

Framing narratives serves the purpose of simplifying intricate matters, making them easier for audiences to grasp by highlighting particular aspects of the content to match audiences’ pre-established mental frameworks [43]. Complex issues like the opioid crisis in the U.S. could demand intricate and instructive narratives that may diverge from TV series’ purpose of entertaining viewers.

Studies in the field of narrative research, with a particular focus on health communication, have consistently demonstrated the significance of analyzing media narratives such as TV series [84], especially in today’s digital age, where TV series have become a dominant form of entertainment and storytelling [85]. Delving into these narratives provides a more profound insight into intricate societal matters and underscores the crucial role narratives play in fostering knowledge, attitudes, and health-related behaviors [84].

While it is not our intention to propose contributions regarding framing conceptualization, we would like to address some theoretical implications of this study. The lack of a cohesive theory and methodology for framing in communication research, what Entman [41] referred to as a “fractured paradigm”, has resulted in both the overuse and misuse of framing [86], thus making it challenging to clearly differentiate framing from other concepts in communication research [87]. However, as pointed out by Ardèvol-Abreu [88], not everyone considers the varied approaches to framing a drawback. D’Angelo [89] suggests that the diverse array of approaches is necessary to comprehend a phenomenon of great complexity like the media, and Reese [90] posits that the significance of framing theory does not reside in its potential as a unified research paradigm, but rather in its ability to bridge the gap between qualitative and quantitative, empirical and interpretive, psychological and sociological, as well as academic and professional research.

Framing can be employed both methodologically and theoretically [91]; however, previous research has used only one or the other approach. Methodological approaches have yet to be standardized [47]. Theoretical approaches have used framing as a theoretical background to interpret the content of news media or fictional narratives. Much of this research has focused on framing news in the media [90, 92]; however, Scheufele [43] clarifies that study frames apply not only to news media but also to journalistic stories across different media, such as print and television. For example, Whiteman et al. [93] analyzed the coverage of scientific articles in news media that showed a possible scientific explanation for breast cancer, focusing on scientific accuracy. Our study also delves into the scientific accuracy of the events depicted in the TV series, comparing them with the existing literature on the opioid crisis.

One of the most original aspects of our research is the exploration of the articulation between narrative analysis and framing. Narrative analysis delineates qualitative narrative threads and analyzes specific themes, characters, and stories according to an objective and well-justified methodology. Framing also encompasses these prominent themes, but it goes further in attempting to reach a broader understanding of reality. Few studies have explored this articulation in depth. Although Listyani et al. [94] used framing to analyze the scripts of Japanese and American cartoon movies, Ye et al. [95] studied the medical frame on the portrayals of illnesses and diseases in two medical dramas, and Wang and Parris [48] used framing theory in the literature review in their analysis of the TV series *13 reasons why*, all these papers used framing theory only as a theoretical background. In their study analyzing the causes and solutions of obesity as portrayed in newspapers and TV news, Kim and Willis [49] took this approach one step further by analyzing the framing of the representation of responsibility in these media. Our research forges ahead on the path these authors laid, focusing on the way *Dopesick* emphasizes different viewpoints to establish a frame of reference (i.e., reference framing) [86] to explain the opioid crisis. This approach makes it clear that the dominant perspective in *Dopesick* ascribes blame to the pharmaceutical company and regulatory organisms, while fostering empathy toward opioid users and their families and championing individuals and organizations who strive to bring the culprits to justice and alleviate the victims suffering.

Bulck et al. [50] combined quantitative content analysis and qualitative framing analysis, providing enhanced insight into thematic elements in their research into the framing of alcohol in a TV series targeting teenagers. Unlike these authors, who were able to rely on codes from prior investigations, we considered it necessary to develop new codes suitable for the opioid crisis. To this end, we used narrative analysis to examine each scene objectively to substantiate the frequency of characters and narrative themes, and we complemented this approach with framing analysis for theoretical and methodological validation.

Furthermore, framing goes beyond narrative themes, inviting us to analyze the manner in which the story is told, its significance, how protagonists and antagonists are constructed, and the narrative aspects of the story. It prompts us to consider from which perspective the story is being narrated and how “that” reality is being constructed.

Regarding methodology, choosing a single TV series for analysis allows for a more in-depth and focused exploration of its narrative elements. By concentrating on one show, we can delve deeply into its characters, plot development, and themes, gaining a comprehensive understanding of its narrative. We can also debate whether the unit of analysis for a narrative analysis should be the scene or the overarching story. We opted for the scene to gain a nuanced perspective on narrative themes, considering both their depth of portrayal and their placement within the overall plot. Moreover, the challenge in conducting such analyses always lies in achieving objectivity and ensuring replicability in subsequent studies, a matter that is more readily defined when segmenting by scenes rather than overarching plots.

From a practical point of view, our analysis can help people understand *Dopesick’s* potential to raise awareness about social perceptions of the people affected by the opioid crisis and about social condemnation of the Sackler family and Purdue Pharma [69, 82]. *Dopesick* might also influence how healthcare professionals, law enforcement agencies, and legal experts perceive and treat patients struggling with substance dependence [96, 97], favoring viewing these individuals within the context of their complete addiction narratives rather than solely through the lens of criminality [98]. It can encourage healthcare providers to adopt a more holistic and compassionate approach, recognizing that opioid misuse or abuse is a complex issue often rooted in various personal, social, and medical factors. These issues remain especially important considering the ongoing crisis involving fentanyl and tramadol [9, 98].

### Limitations of the study and future directions

Some limitations of our study require comment. While *Dopesick* offers valuable insights into the opioid crisis, it focuses on specific characters and situations and largely ignores or deemphasizes other elements and characters that played an important role in the crisis. This was to be expected, because the opioid crisis was a complex phenomenon that cannot be fully encapsulated by any single TV series or movie. Time constraints, narrative structure, and commercial considerations inevitably shape the portrayal of complex events in TV series. Given the finite number of episodes and runtime, the series had to prioritize certain aspects of the crisis over others, potentially oversimplifying or omitting critical dimensions in striving to engage audiences and maintain viewer interest. However, all approaches to portraying complex situations are limited in different ways, and examining the frames and perspectives in which the events are narrated can shed new light on the situation. *Dopesick* serves as a compelling entry point to raise awareness about the opioid crisis, and the series provides much useful material for discussion and understanding. Nevertheless, it cannot provide a comprehensive understanding of the multifaceted factors contributing to the crisis.

Thus, the series and our analysis cannot hope to provide a comprehensive view of the opioid crisis, but it may help us understand how the narrative deals with (or fails to deal with) elements besides Purdue Pharma that were involved in the crisis, such as the healthcare system, other drug companies, or the press. Certainly, future studies can be conducted on other narratives about the opioid crisis in the U.S.; it would be interesting to see whether they select and highlight the same themes or if different themes emerge. These studies can examine fictional narratives, documentaries, or journalistic portrayals in news media, as well as comparing storytelling in different approaches. It could also be interesting to for new studies to use Fisher’s concepts of fidelity (and even coherence) to compare *Dopesick* with other TV series (e.g., Netflix’ *Painkiller*); such studies could also examine viewers’ experiences, perhaps employing focus groups with audiences from different backgrounds.

Our study focuses on media frames rather than audience frames [37], because we were interested in studying how the reality of the opioid crisis is depicted in the TV series *Dopesick*, rather than how the audience accepted or rejected this narrative. Specifically, our emphasis is on examining how the narrative of the opioid crisis is constructed within the TV series; future research might analyze the series’ impact on the audience. It is important to explore whether these narratives have an impact on public opinion or people’s attitudes toward these health-related topics, since health narratives can help people gain a deeper understanding, develop emotional connections, and ultimately enhance well-being while promoting greater empathy towards others [81]. Future studies might gauge the short- and long-term effects of this TV series on viewers’ knowledge and attitudes.

New studies should also consider the actual value of *Dopesick* for understanding the most important elements that contributed to the crisis. The series promises to be useful in teaching various disciplines (e.g., health sciences, law, and sociology), and studies collecting empirical data about the effectiveness of activities based on *Dopesick* in increasing students’ knowledge and understanding would be useful and would enhance their educational value.

## Conclusions

*Dopesick* is the first TV series centered on the opioid crisis in the U.S. Although the series shows the crisis from different perspectives and reveals multiple dimensions in its storytelling, it nevertheless downplays the roles of many agents and focuses on the Sackler family in general and Richard Sackler in particular as the cause of the problem. There is a general tendency to favor simple explanations and simple solutions to complex problems, such as the narrative of overprescribing opioids sparking a public health crisis in the United States [77], and *Dopesick* fails to avoid this pitfall. Rather than provide a nuanced analysis of a complex crisis, the series puts the blame almost entirely on OxyContin^®^ and Purdue Pharma.

Narratives provide a potent avenue for understanding, communicating, and gaining insights from personal experiences of illness and recovery [81]. In this sense, *Dopesick* may foster empathy towards substance-dependent individuals. On the other hand, it has the potential to introduce the topic of the misuse of prescription opioids, as it reinforces its connection with addiction and stigmatized beliefs regarding irrationality and lack of control [69, 99].

*Dopesick* provides viewers with an initial framework for sharing and discussing inappropriate opioid use in general, beyond Purdue Pharma. Indeed, the series is innovative and useful, bringing an important topic to open television and making it possible for the public to learn about and discuss science, pharmaceutical companies, regulatory agencies, health institutions, and even stereotypes of Appalachians.

## Supporting information

S1 Annex Chronological scheme of the events shown in the series *Dopesick* according to the episodes and the main characters. (DOCX)

S1 Data set (XLSX)
