# Supplementary material for: Telling the story of the opioid crisis: A narrative analysis of the TV series Dopesick
Source: PLoS One. 2024 Apr 4;19(4):e0301681. doi: 10.1371/journal.pone.0301681 (PMC10994355; doi:10.1371/journal.pone.0301681)
Supplement: S1 Annex — (DOCX) [file pone.0301681.s001.docx]

**Annex 1**: Chronological scheme of the events shown in the series *Dopesick* according to the episodes and the main characters

|  | Episode 1  *First Bottle* | Episode 2  *Breakthrough Pain* | Episode 3  *The 5th Vital Sign* | Episode 4  *Pseudo-Addiction* | Episode 5  *The Whistleblower* | Episode 6  *Hammer the Abusers* | Episode 7  *Black Box Warning* | Episode 8  *The People vs. Purdue Pharma* |
| --- | --- | --- | --- | --- | --- | --- | --- | --- |
| Richard Sackler (Purdue Pharma) | 1996 OxyContin Launch | 1996  Breakthrough pain concept proposal  1996 Sales improvement  1996 Concern about increasing the dose | 1996 Proposal to make pain “the fifth vital sign”  1996 Individualizing dosage and treating breakthrough pain before it occurs  1996 Wish to export OxyContin to Germany | 1997 Arthur Sackler inducted into the Medical Advertising Hall of Fame  1997 Hiring of David Haddox | 1999 In two years sales tripled; 160mg tablet introduced.  1999 Named President of Purdue Pharma  1999 Purchase of the building in Stamford | 2000 Concern over the investigation led by Jay McCloskey, the U.S. Attorney for Maine  2000 Purdue Pharma executives meet with McCloskey | 2001 Concern over FDA label review  2001 FDA adds black box warning but allows sale for moderate pain and for an extended period of time | 2002 Purdue Pharma’s executives meet with Dr. Art Van Zee.  2002 Michael Friedman President of Purdue Pharma  2019-2020 Documentary images of the declarations in US Congress |
| Billy Cutler (Purdue Pharma sales rep) | 1996 OxyContin sales rep training begins | 1996 The “breakthrough pain” concept is presented to sales reps  1996 Purdue Pharma’s seminar in Arizona. Billy introduces Dr. Finnix  1996 Sales reps double their salaries by doubling prescription doses  1996 The 80 mg pill is introduced | 1996 Training on “individualizing dosage”  1996 “Toppers Contest”  1996 Advises Dr. Finnix that he can stop the medication without lowering the dose and without fear of withdrawal symptoms | 1997 Training on “pseudoaddiction” with David Haddox  1997 The personal relationship with Dr. Finnix is broken | 1999 Observation of OxyContin snorting  1999 Visits Dr. Finnix at the rehab clinic | 2000 Apathy in celebrating sales reps | 2002 Apathy in training | 2002 Theft of Purdue Pharma training videos; gets fired.  2006 Meeting with prosecutors: He denies having the videos, but the videos later arrive at the prosecution |
| Rick Mountcastle / Randy Ramseyer (Assistant U.S. Attorneys for Virginia) | 2002 Investigation starts: DEA brought in, they suspect the label and note that Curtis Wright (FDA) was hired by Purdue Pharma.  2002 They study the advertising video of the drug “I got my life back”  2005 They take statements from doctors | 2005 They take statements from doctors  2003 OxyContin video investigation: interviews with the ad agency, Dr. Alan Spanos, and patients appearing in the video  2003 Meeting with the FDA: FDA explains they lack the staff to oversee drug ads. Proposal to charge Purdue Pharma with criminal misbranding. | 2003 The judge rejects the request for Purdue’s internal information materials because he considers that the demand is too broad  2003 Ramseyer undergoes surgery and is offered OxyContin at the hospital. Both question the hospital staff about this offer.  2003 Purdue's connections to pain and patient associations are established. With this proof, the judge requires Purdue to provide the information. | 2004 Statements  2004 They investigate which study the OxyContin advertising phrase (< 1% become addicted) was based on. They find the published paper and summon its author (Dr. Herschel Jick) to testify in court. | 2004 Funding for the investigation increases and the team expands  2004 In an interview a Purdue Pharma sales rep confirms that the abuse of the drug was known; she does not want to testify in court because she signed a confidentiality clause.  2004 Contact and interviews with Maryanne Skolick  2004 Interview with Maureen Sara and confirmation that Purdue was aware of the abuse. Sara became addicted to OxyContin and has a stress relapse. She can't testify at trial. | 2005 They investigate a graph of the absorption of OxyContin used in promoting the drug, finding that it was deceptive, and incomprehensible that the FDA approved it.  2005 They try to contact McCloskey to no avail. He was hired by Purdue Pharma. | 2006 They investigate how the label was approved by the FDA  2006 They charge Purdue Pharma with lying to Congress and conspiring to defraud  2006 They receive pressure for abandon the case | 2006 They charge Purdue with wire fraud and conspiracy to commit wire fraud.  2006 They interview the head of training at Purdue Pharma, who confirms the theft of the videos.  2006 Videos of Purdue Pharma trainings arrive at the prosecutor's office.  2006 Draft of the indictment: conspiracy to commit mail fraud, wire fraud, interstate distribution of a mislabeled drug with intent to defraud, conspiracy to commit money laundering, and money laundering.  2006 Executives agree to plead guilty and pay $600 million, the largest settlement in US history  2007 Settlement in Abingdon |
| Bridget Meyer (DEA) | 1999 Beginning of investigation into the illegal commercialization of OxyContin due to the increase in crimes  1999 She learns from the FDA that the person who approved the OxyContin label was Curtis Wright, who now works for Purdue Pharma | 1999 In independent research at a pain clinic in Kentucky, she interviews a teenager who tells her about the use/diversion of OxyContin. | 1999 Thefts from pharmacies continue. The pharmacist tells her that he cannot stop selling OxyContin because the sales rep has threatened him. The pharmacy robber dies of an overdose.  1999 She is transferred to Diversions, becoming the Deputy Director | 2000 She meets with the FDA. They refuse to impose restrictions without proof that the drug is dangerous.  2000 She meets with Purdue Pharma but the company denies the risks of the drug | 2001 Press conference to pressure Purdue Pharma  2001 She meets with Purdue Pharma and Richard Sackler, but reach no agreement is reached | 2002 She meets with the FDA again. The FDA holds that OxyContin is safe when used as prescribed. Agent Meyer agrees to prove that the drug is not safe.  2002 DEA investigation of OxyContin overdose autopsies following prescription  2002 Meeting with Rudy Giuliani, who will represent Purdue Pharma  2002 News of the death of the young man she interviewed in 1999 | 2002 She gives the investigation report to the press before speaking with the FDA  2002 DEA-FDA-Purdue Pharma meeting at the FDA: 98% of the dead were not drug abusers; FDA says the data is inconclusive. |  |
| Dr. Samuel Finnix | 2005 Testifies in the trial against OxyContin  1996 Begins prescribing OxyContin after hosting sales rep Billy Cutler | 1996 He prescribes OxyContin to Logan Parker, the pain goes away  1996 Purdue invites him to a seminar in Arizona, where he meets Dr. Russel Portenoy, a prestigious researcher. Dr. Finnix is persuaded to make a presentation about his patients and the use of OxyContin. | 1996 He is invited to Orlando by Purdue  1996 After an automobile accident, he receives OxyContin for the pain (20mg). | 1997 Logan Parker complains that he feels numb. Dr. Finnix lowers his dose from 80mg to 40mg.  1997. Dr. Finnix has severe symptoms of dependence. He takes medication belonging to his patients; he asks a Purdue executive for samples and hides medication in his house.  1997 He hits Billy Cutler and tells him that he "sells poison"  1997 He buys OxyContin from a drug dealer, who recommends snorting it. | 1999 He asks for OxyContin prescriptions in different towns. He’s taking 400mg a day.  1999 He loses control in a surgical procedure, is placed on probation, loses his medical license, and must do rehab for 90 days.  1999 He gets a visit from Billy Cutler at the rehab clinic and asks him for OxyContin. | 2000 He participates in treatment groups at the rehabilitation center.  2000 He finishes 90 days of rehabilitation and when he returns home, he relapses.  2000 He starts treatment with liquid methadone (35mg). He must go to a methadone distribution center every day to take the medication. | 2002 He continues methadone treatment and does psychotherapy with Sister Beth Davies. He wants to regain his medical license, but learns it is impossible as long as he is on methadone  2002 Van Zee recommends that he take Suboxone | 2002 He is treated with Suboxone treatment  2002 He agrees to take one of his patients to treatment. He buys a bus to take more people.  2021 He runs a rehab clinic in town |
| Betsy Mallum | 1996 She is injured at work in the mine; Dr. Finnix prescribes her OxyContin (10mg) | 1996 She wakes up in pain in the morning; Dr. Finnix doubles her dose (20mg)  1996 Her dose is doubled again (to 40mg); she has the first symptoms of dependence on OxyContin | 1996 After four weeks of treatment, the doctor lowers her dose to one pill per week. Betsy has withdrawal symptoms.  1996 Working in the mine with withdrawal syndrome, she causes an explosion | 1997 Now she works at a gas station, with withdrawal symptoms: sweating, nervousness, tics.  1997 She agrees to go to a pain clinic in Florida with her drug dealer, where the doctor offers her to exchange sex for his fee, but she doesn't accept  1997 She goes with her mother to an addicts meeting. One of the members of the group sells her pills in the bathroom. | 1999 She pawns her mother's jewelry. She agrees to have sex in exchange for more money.  1999 She enters a rehabilitation clinic where they bind her hands and feet. | 2000 She participates in church ceremonies to fight her addiction; she take up sports. She is seen having withdrawal symptoms.  2000 She looks for OxyContin, and tries heroin. | 2001 She gets high with heroin; her mother goes looking for her  2002 With her mother, she attends a meeting with Dr. Art Van Zee and Sister Beth Davies, where ten thousand signatures are collected to request that OxyContin be withdrawn from the market.  2002 She accepts Dr. Finnix’s proposal to start treatment with Suboxone, but she seeks one more heroin high "as a farewell" and dies of an overdose. | 2002 Betsy’s father’s alcoholism  2019 Her mother participates in the "Shame on Sackler" demonstrations |
